# Validation of an MRI Technique for the 6-DOF Knee Kinematics Measurement

**DOI:** 10.3389/fbioe.2022.904012

**Published:** 2022-09-14

**Authors:** Shixiong Tang, Liwen Zheng, Yongheng Luo, Ren Wu, Qunyan Tian, Lei Wang

**Affiliations:** ^1^ Department of Radiology, The Second Xiangya Hospital, Central South University, Changsha, China; ^2^ Clinical Research Center for Medical Imaging in Hunan Province, Changsha, China; ^3^ Department of Rehabilitation, The Second Xiangya Hospital, Central South University, Changsha, China; ^4^ Department of Orthopedics, The Second Xiangya Hospital, Central South University, Changsha, China; ^5^ School of Information Science and Engineering, Central South University, Changsha, China

**Keywords:** total knee arthroplasty, knee joint kinematics, prosthesis design, characteristic area, characteristic area frame, subcortical vascular segment, most stable rotation axis

## Abstract

**Background:** For total knee arthroplasty (TKA), the optimal rotational position of the femoral component is felt to be critically important. The current knee joint kinematics measurement technology is unable to identify the exact rotation axis of the knee joint, the main reasons being low measurement accuracy and insufficient three-dimensional data (2D-3D image matching technology). In order to improve the effect of TKA surgery, we proposed a knee joint kinematics measurement method, based on the MRI technology, and verified its measurement accuracy. We then employed this method to identify the personalized optimal rotation axis of the knee joint for TKA patients.

**Purposes:** The purpose of the study was 1) to propose a method for measuring knee joint kinematics and verify its accuracy and 2) to propose a method for determining the optimal rotation axis of knee joint for TKA surgery, based on accurate kinematic measurement results.

**Materials and Methods:** The experiment was divided into two parts: *in vitro* and *in vivo*. The purpose of the *in vitro* experiment was to verify the measurement accuracy of our method. We fixed two aquarium stones (approximately 10 cm * 10 cm * 10 cm in size, close to the size of the distal femur and proximal tibia) firmly on the fixed and moving arms of the goniometer/vernier caliper with glue and immersed the aquarium stones in the water to capture MRI images. The MRI images were then processed with MATLAB software, and the relative motion of the two aquarium stones was measured. The measurement accuracy of our method was verified *via* the scale reading of the moving arm on the goniometer/vernier caliper. *In vivo*, 36 healthy elderly participants (22 females, 14 males) were recruited from the local community; our method was then employed to measure the relative motion of the tibia and femur and to observe the rollback and screw home motion of the medial/lateral condyle of the femur, which was identified as specific kinematic features of the knee joint.

**Results:** *In vitro*, all measurements were accurate to <1 mm and <1°. *In vivo*, all knee measurements showed rollback motion (the rollback distance of the medial femoral condyle was 18.1 ± 3.7 mm and that of the lateral condyle was 31.1 ± 7.3 mm) and screw home motion.

**Conclusion:** In the application scenario of knee joint kinematics measurement, our method has an accuracy of <1° of rotation angle and <1 mm of translation for all reference points, and it can be employed to identify the most stable axis of the knee joint.

**Significance:** Using our method to accumulate data on the knee rotation axis of more subjects to establish an average rotation axis of a given population may help in knee prosthesis design and reduce the patient dissatisfaction rate. Individually measuring the patient’s rotation axis before TKA surgery and adjusting the prosthesis installation in TKA may further reduce the patient dissatisfaction rate, and automatic computer measurement may be realized in the future, but it is still time-consuming for now.

## Introduction

Total knee arthroplasty (TKA) is a commonly used surgery that treats end-stage osteoarthritis (OA). It is estimated that by 2030, the number of patients undergoing TKA in the United States will reach 3.5 million every year (Kurtz et al., 2007). So far, 8%–25% of patients undergoing TKA are dissatisfied with the outcome of the surgery (Justin et al., 2012; Baker et al., 2013; Dunbar et al., 2013; Schulze and Scharf, 2013). In order to improve the efficacy of the surgery, researchers performed numerous kinematics researches, in recent decades, focusing on the rotation axis, which is felt to be critically important for TKA, and the design of the knee joint prosthesis components depends on the assumption of the correct rotational axis.

To accurately locate the rotation axis, a simple and effective method for describing the relative motion between the femur and tibia is required. It is easy to imagine that the relative motion of two rigid bodies refers to the change of orientation and displacement of one body (the moving rigid body, the femur is used as the moving rigid body in this experiment) at different time points, with the other body (the fixed rigid body, the tibia is regarded as the fixed rigid body in this experiment) as a reference. In the actual measurement, the mutual position of the two rigid bodies at a certain moment can be defined as the initial state of motion (in this study, the knee joint was extended as the initial state), and one or more terminal states of motion can be determined (different angles of knee joint flexion), then the orientation and displacement changes of the moving rigid body (femur) can be measured in each terminal state, relative to the initial state, and thus, these changes can be quantitatively described. In recent studies, researchers generally described knee kinematics using 6 degrees of freedom (DOF) (Li et al., 2009; Fitzpatrick et al., 2016; Guan et al., 2017; Sta et al., 2021). Although the details of these descriptions differ, the principle of the rigid body rotation with 3 DOF (3 rotation directions in 3-dimensional space) and rigid body displacement with the other 3 DOF (3 coordinate axes in 3-dimensional space) remain the same. Reddy et al. established a description method of 6-DOF. They believed that the most important aspect was to determine the 3 rotational DOF. Upon determining the rotation angle, the measurement and calculation of the 3 translational DOF would be very simple (Reddy et al., 1989).

In addition to the describing method, the precise recording of the moving process is also critical. In order to examine the rotation axis, many studies selected the contact point (Mohsen et al., 2016; Cacciola et al., 2020; Takashi and Mochizuki, 2021) or reference point (Meneghini et al., 2017) to be the measurement object. Freeman and Pinskerova (2005) believed that the contact points are not suitable for analyzing the axis of rotation, because they do not define a point in the femur. Skin sensor (Schache et al., 2006; Van Campen et al., 2011) has low accuracy due to soft tissue movement (Dennis et al., 2005; Freeman and Pinskerova, 2005; Beach et al., 2019). In addition, there are substantial individual differences in the measurement of bony eminence (Şahin et al., 2013).

In addition, non-invasive measurement methods are more easily accepted by patients and applied in the clinic. For example, the cortical bone screw has high accuracy, but it is invasive (Ramsey and Wretenberg, 1999), so it is generally used only in cadaver studies. Imaging examinations (X-ray, CT, MRI) are non-invasive, but the radiation dose of CT is relatively large. In the scenario of kinematic measurement that requires repeated CT scans of the knee joint, the potential health risks of experimental subjects/clinical patients are particularly worrying. X-ray is less radioactive, and 2D-3D image matching technique is developed, based on X-ray fluoroscopy, whose accuracy is very high (Giphart et al., 2012; Kono et al., 2018). But they have a common deficiency: Only the reference points with local features (e.g., metal beads and contact points.) can be tested to validate the translation accuracy (Yamazaki et al., 2015; Shih et al., 2020). It cannot validate the translation accuracy of other reference points without local features. This deficiency can be made up for with weight-bearing MRI techniques, which are less popular but still an active research area (Bruno et al., 2018). Some researchers employed MRI to measure the knee joint kinematics (Hollister et al., 1993; Scarvell et al., 2004; Park et al., 2014; Dzialo et al., 2018; Yu et al., 2021) but the accuracy was not very good. We believe that the main obstacle is the lack of a reference object that can be accurately measured and widely used. The main innovation of our study is the use of the subcortical vascular segment (SCVS) as the universal reference. We use multiple (6–8 each in sagittal plane/coronal plane/horizontal plane, 18–24 in total) SCVS to create a large (length/width/height>4 cm) characteristic area frame (CAF), which is then used to locate the reference points on the femur/the tibia. Because of their small size and distinctive features, SCVS are more accurately located than traditional anatomical markers, such as tibial tubercle.

Our purpose is 2-fold: 1) To propose an MRI-based non-invasive *in vivo* knee kinematics measurement method and to verify its measurement accuracy; 2) to describe a method for determining the optimal rotation axis of knee joint for TKA surgery, based on accurate kinematic measurement results, and discuss the logic of this method. Our assumption is that the measurement accuracy of this kinematics measurement method is <1 mm and <1°.

## Materials and Methods

**FIGURE 1:**
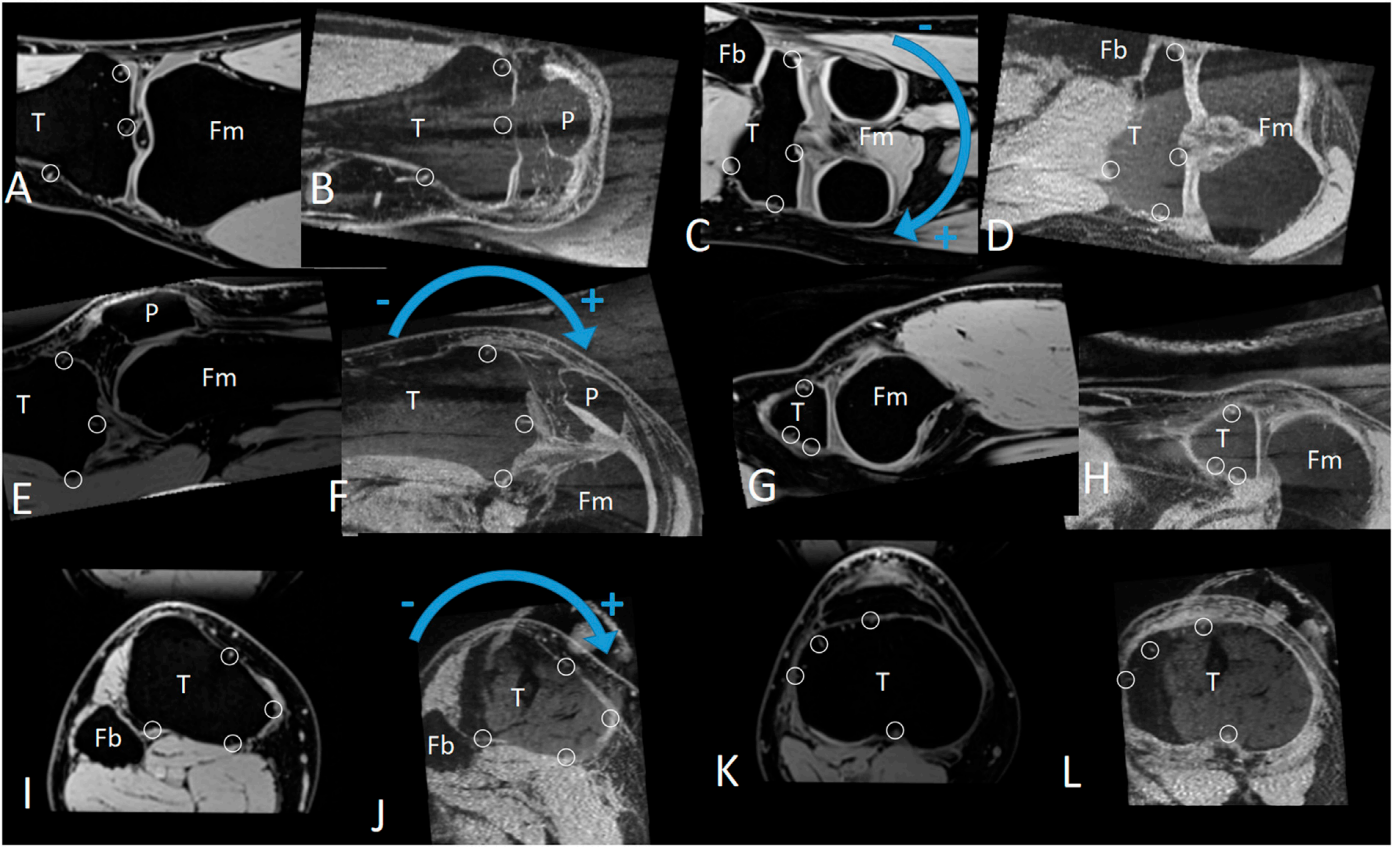
The positive and negative directions of the coronal, sagittal, and horizontal plane rotation, as well as the characteristic area frame (CAF, composed by multiple characteristic areas). **(A–D)** Coronal plane. **(E–H)** Sagittal plane. **(I–L)** Horizontal plane. Small circles: Subcortical vascular segment(SCVS) selected as the characteristic area. T: tibia. P: patella. Fm: femur. Fb: fibula.

This study employed the geometric center axis (GCA) coordinate system (Most et al., 2004; Eckhoff et al., 2007; Iranpour et al., 2010; Van Campen et al., 2011). The 3-dimensional rotation of the bone was described by the Carden angle (Tupling and Pierrynowski, 1987). The rotational order was: sagittal plane to horizontal plane to coronal plane. The rotation unit was degree, with positive numbers representing backward/internal rotation/valgus, and negative numbers representing forward/external rotation/varus (Figure 1).

### Principles of Measurement and Description for Relative Motion

**FIGURE 2:**
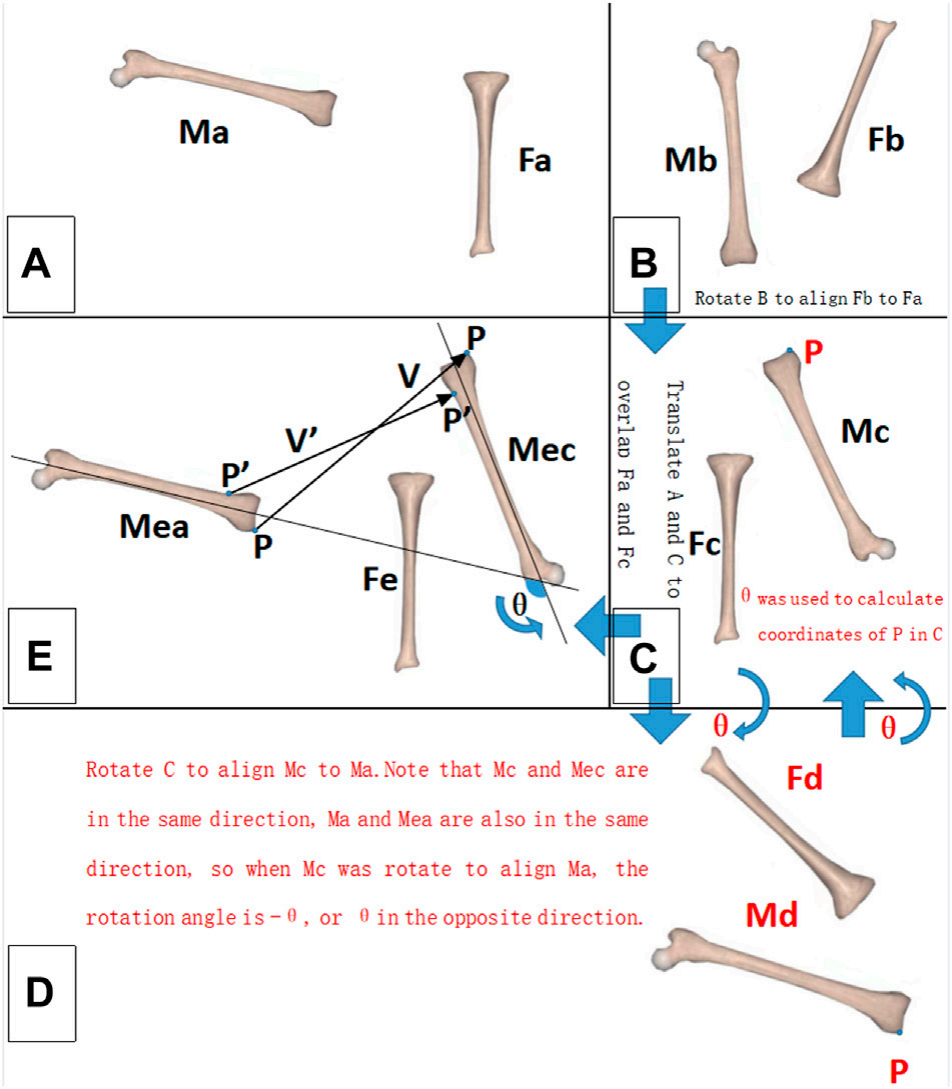
Schematic diagram of the relative motion measurement. For a 2-dimensional rigid body, the black font part should be noted; for a 3-dimensional rigid body, the black and red font parts should be noted. **(A)** Initial state of the rigid bodies. **(B)** Terminal state of the rigid bodies. **(C–E)** Measurement process. M: moving rigid body. F: fixed rigid body. P, P′: reference point. V, V′: displacement vector of the reference point. *θ*: rotation angle of the moving rigid body (M).

To help understand our method of measuring relative motion, let’s first look at the case of a 2-dimensional rigid body (Figure 2, black text). The relative motion between the fixed rigid body F and moving rigid body M can be described *via* two parameters: 1) the rotation angle θ of M 2) the displacement vector V of any reference point P on M. Since the entire 2-dimensional rigid body can be easily visualized to the image, we can measure the rotation angle θ and the displacement vector V directly on the image. Obviously, V is different for different reference points P. If the displacement parameter V of P is measured, the displacement parameter V′ of any other reference point P′ can be calculated by using relative position P′-P (Eq. 1).

$${\text{V}}^{\prime }=\left({\text{P}}^{\prime }-\text{P}\right)\left[\begin{array}{cc} \cos\mathrm{\theta ,} & -\sin\theta \\ \sin\mathrm{\theta ,} & \cos\theta \end{array}\right]+\text{V}$$ (1)

The situation of a 3-dimensional rigid body is similar to that of a 2-dimensional rigid body, except for one troublesome aspect, we cannot see the full picture of a 3-dimensional rigid body in MRI images, so we cannot measure θ and V directly as we can with a 2-dimensional rigid body. We solved this problem by noting that Mea and Ma are in the same direction and Mec and Mc are in the same direction. As long as we rotated A so that Ma and Mc were in the same direction, the rotation operation would be equivalent to θ, or if we rotated C so that Mc was in the same direction as Ma, this rotation operation would be equivalent to −θ (Figure 2, red text section). The measurer tried different rotation operations. Given the directions of Ma and Mc are in the same direction, the current rotation operation will be the measurement result of θ. The measurement accuracy of this method depended on the accurate determination that Ma faced the same direction as Mc after the rotation operation. To improve the accuracy, we innovatively proposed the concept of the CAF (composed by multiple SCVS) as a tool to confirm the same direction of two identical rigid bodies (Figure 1). After determining θ, V can be directly measured, because after the θ rotation operation (with P as the rotation center) the CAF of Mea is parallel with the CAF of Mec (on MRI images the 2 CAFs are identical), so V is the positional difference between the two parallel CAFs. The displacement parameter V′ of any other reference point P′ was then calculated using Eq. 2. In other words, only V was measured. Hence, all V′ was calculated. The measurement accuracies of V and V′ were tested separately, as described in the “*In vitro* measurement accuracy test” section.

$${\text{V}}^{\prime }=\left({\text{P}}^{\prime }-\text{P}\right)\text{RxRyRz}+\text{V,}$$

where

$$\text{Rx}=\left[\begin{array}{ccc} 1 & 0 & 0\\ 0 & \cos a & -\sin a\\ 0 & \sin a & \cos a \end{array}\right]$$

$$\text{Ry}=\left[\begin{array}{ccc} \cos b & 0 & \sin b\\ 0 & 1 & 0\\ -\sin b & 0 & \cos b \end{array}\right]$$

$$\text{Rz}=\left[\begin{array}{ccc} \cos c & -\sin c & 0\\ \sin c & \cos c & 0\\ 0 & 0 & 1 \end{array}\right]$$

$$\text{RxRyRz}=\left[\begin{array}{ccc} \cos b\cos c & \sin a\sin b\cos c+\cos a\sin c & -\sin b\cos a\cos c+\sin a\sin c\\ -\sin c\cos b & \cos a\cos c-\sin a\sin b\sin c & \cos a\sin b\sin c+\sin a\cos c\\ \sin b & -\sin a\cos b & \cos a\cos b \end{array}\right],$$ (2)

θ=[a,b,c]

Regarding Eq. 2, the rotation matrix in our formula was the same as in the study of Reddy et al. (1989). We divided the rotation matrix into three matrices, representing the rotation of the sagittal, coronal, and horizontal planes. This did not affect the calculation results; however, it made the formula more readable.

In the 3-dimensional situation, θ and V had 3 parameters each, for a total of 6 DOF. Although P also had 3 parameters, P was determined by V using Eq. 2, so the parameters of P were not free. In other words, the method we used to describe relative motion had 6 DOF.

### *In Vitro* Measurement Accuracy Test

**FIGURE 3:**
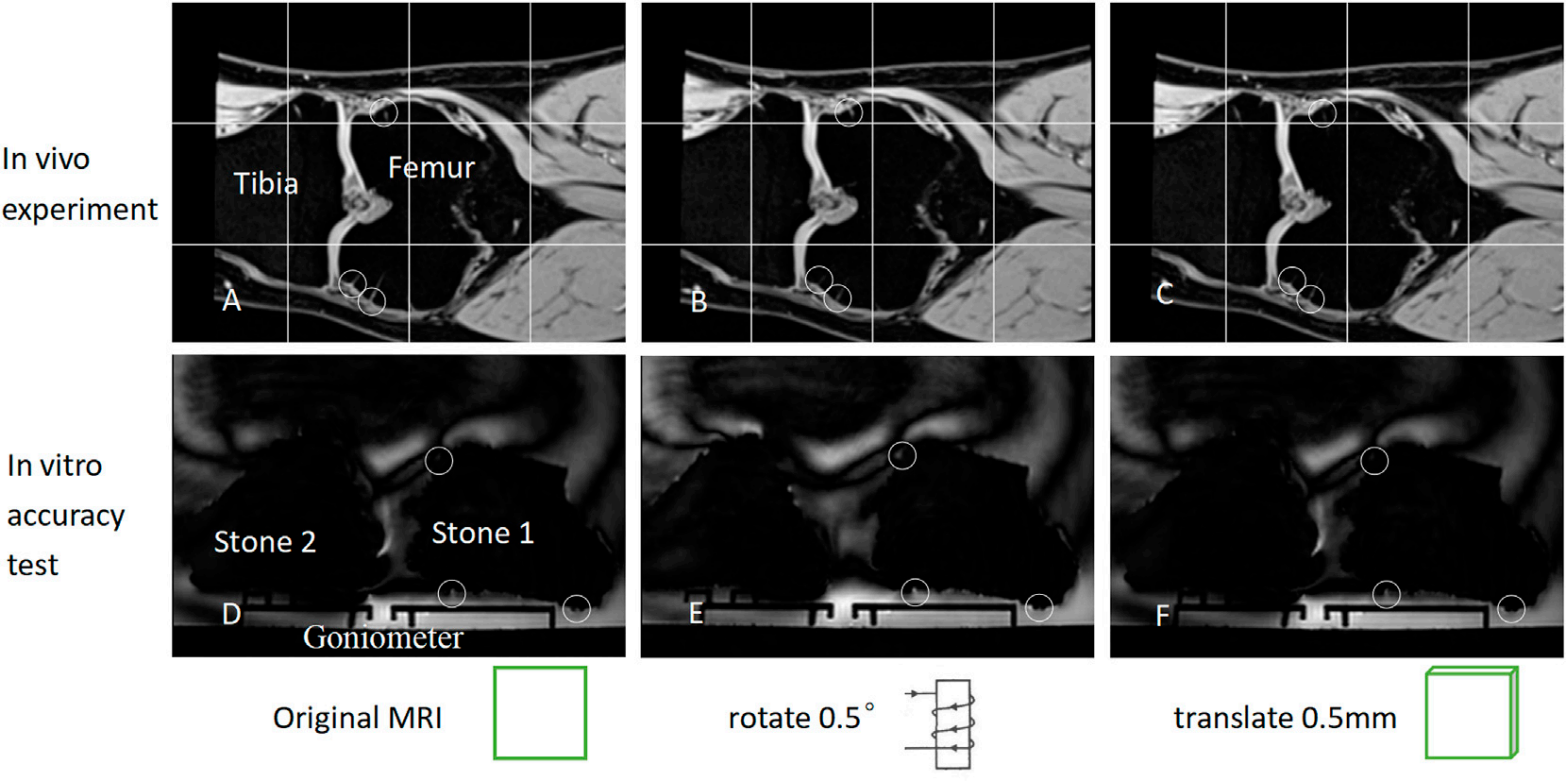
**(A)** depicts the coronal MRI of the knee joint prior to rotation/displacement. It should be noted that the SCVS is marked with small circles. **(B)** illustrates the coronal plane image following rotating **(A)** in the sagittal plane by 0.5°. It should be noted that the SCVS has altered. **(C)** depicts the image obtained by displacing **(A)** by 0.5 mm perpendicular to the coronal plane. It should be noted that the SCVS also alters. As long as one characteristic area (SCVS/surface indentation) is different, it can be determined that the rigid body has either undergone a slight rotation or a slight translation. **(D–F)** is similar to **(A–C)**, except that the tibia and femur changed into aquarium stones. A rotation of 0.5° or a displacement of 0.5 mm can, therefore, alter the characteristic area significantly.

Since the CAF (Figure 1) generally has a significant impact on measurement accuracy, we set a unified standard for it in this study: 1) The length/width of each characteristic area(SCVS/surface indentation of aquarium stones) was ≤5 pixels (2.5 mm); 2) The characteristic areas were distributed in two different MRI planes, with ≥80 layers (4 cm) interval between the planes in the direction of each coordinate axis; and 3) When comparing the similarity between the CAF of both rigid bodies, each characteristic area as well as the interval between the layers where the characteristic area was located were made the same. Under such a standard, CAF is sensitive to a small rotation of the rigid body, and a 0.5° deflection in the direction of the rigid body can cause a visible change in the CAF (Figure 3). If the same CAF can be found on two rigid bodies, it means that the two rigid bodies are identical and have little difference in direction. Therefore, depending on CAF, it can be determined that the direction of the moving rigid body in the terminal state after rotation is the same as that of the moving rigid body in the initial state, thus accurately measuring the rotation parameter θ (as described in the “Principles of measurement and description for relative motion” section mentioned earlier). Similarly, the standardized CAF is also sensitive to a small displacement of the rigid body. In fact, 0.5 mm displacement of the rigid body can cause a significant alteration in CAF (Figure 3), so the displacement parameter V can also be accurately measured.

A two-arm goniometer was used to measure rotation angle, and a vernier caliper represented the length. The angle and length were measured and tested on the sagittal, coronal, and horizontal planes.

**FIGURE 4:**
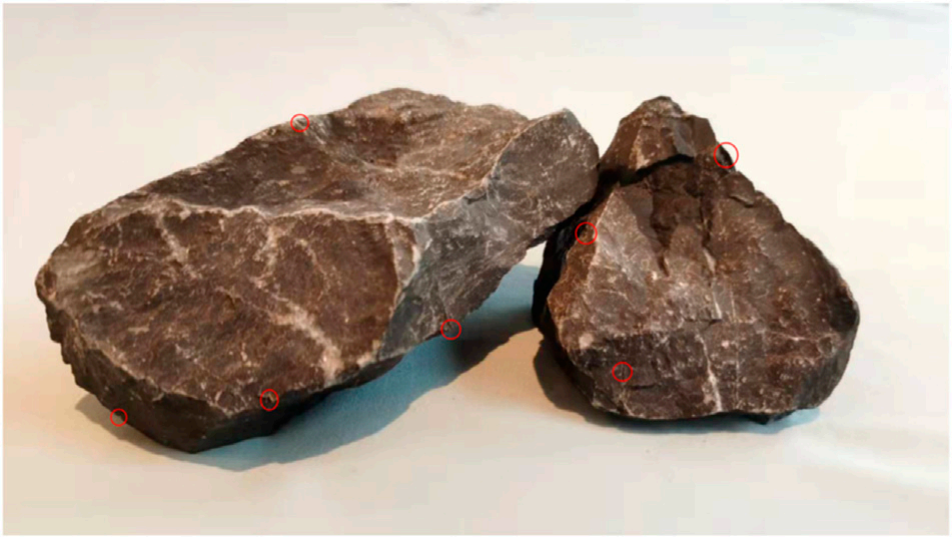
There are many small indentations on the surface of the aquarium stones (small red circles). In their MRI image, those indentations that meet the CAF standard are used as characteristic areas, to improve the measurement accuracy.

Aquarium stones (approximately 10 cm * 10 cm * 10 cm in size, close to the size of the distal femur and proximal tibia) were employed as the rigid bodies to be examined. The exact size of aquarium stones was unknown and their shape was not regular, but this did not affect the measurement of the relative motion between the stones, using our method, much like the irregular shape of the tibia and femur. There are a lot of small indentations on the surface of the aquarium stones, which can be used as a measurement reference like SCVS (Figure 4). We employed glue to firmly fix the two aquarium stones on the moving and fixed arms of the goniometer/vernier caliper. The stones have a low signal on MRI. During MRI examination, we soaked it in water, so that the indentations on the surface of aquarium stones were clearly visualized on MRI. These indentations simulated knee joint SCVS in MRI images and played the role of the characteristic area, to improve measurement accuracy.

The parameter settings of the MRI examination were the same as the “*In vivo* experiments” described as follows. An MRI image was captured at the initial motion state of the goniometer 0°/vernier caliper 0 mm (Figure 2A). Next, the moving arm of the goniometer/vernier caliper was moved to different target scales and MRI images were captured again (terminal state of motion, Figure 2B). The relative motion of the 3-dimensional rigid body was measured using the method described in the “Principle of measurement and description for relative motion” section, and the measurement results were subtracted from the scale of the goniometer/vernier caliper to obtain measurement accuracy.

During the test, the rotation measurement accuracy of the entire rigid body was first tested. Since the knee joint flexion angle generally does not exceed 150°, we tested multiple rotation angles within 150°. Next, the displacement measurement accuracy of the reference points at different positions was tested. First, a reference point P was randomly selected in the area near the center and inside the aquarium, stones to measure its displacement parameter V, to test the displacement measurement accuracy of this reference point P. Then, the displacement measurement accuracy of all reference points within a certain range around P (these reference points can be anywhere near P, represented by P′) was tested. Because the size of the skeletal portion of the knee joint is generally no larger than 10 cm * 10 cm * 10 cm, if P is in the center of the knee joint, it is enough to test the displacement accuracy of the reference point P′ within 5 cm around P. Since the reference point P randomly selected during the actual measurement may not be the exact center of the knee joint, to ensure that the validation range includes the entire proximal tibia and distal femur, we extended the test range and tested the displacement accuracy of all reference points P′ within the range of 7 cm around P with 1 cm intervals. (3-dimensional cube grids of 14 cm * 14 cm * 14 cm, grid spacing of 1 cm, and grid intersections were regarded as reference points P′, with a total of 15 * 15 * 15 = 3,375 reference points and 3,374 reference points except for P).

### *In Vivo* Experiments

From December 2019 to February 2021, 36 subjects (22 females and 14 males) with age >50 years were recruited from residential areas around the hospital with random house numbers. The study was reviewed and approved by the hospital Ethics Committee, and all subjects signed informed consent before the initiation of the study.

We employed the method of Gogia et al. (1987) to measure the knee flexion angle involving the greater trochanter of the femur, fibular head, and lateral malleolus as surface markers. The subject laid on his side with the knee joint to be examined above the contralateral lower limb. A customized and MRI-compatible device was used to apply a 15 kg force toward the head side to the sole of the foot of the subject. Following each MRI imaging, the subject rested for 2–3 min prior to the initiation of the MRI imaging of the next knee flexion angle. MRI was conducted on a clinical 3-T (UMR790, United Imaging, Shanghai) that used fat-suppressed T1-weighted 3D sequence: TR/TE 10.44/4.88; echo train length 22; field of view 160 mm * 160 mm; matrix 320 * 320; receiver bandwidth 150; slice thickness 0.5 mm with no gap; at 2 excitations; and examination time 4 min 43 s. 14 MRI images with knee flexion from 0° to 130° were captured at intervals of 10°. All MRI data were exported in DICOM format. The MATLAB software was used to implement the measurement steps mentioned in the “Principle of measurement and description for relative motion” section. SCVS was used as a characteristic area; these structures were common in MRI under the above-mentioned imaging conditions and met the conditions required for accurate measurement (see the “*In vitro* measurement accuracy test” section for details).

### Method to Determine the Optimal Rotation Axis of the Knee Joint for Total Knee Arthroplasty Surgery

**FIGURE 5:**
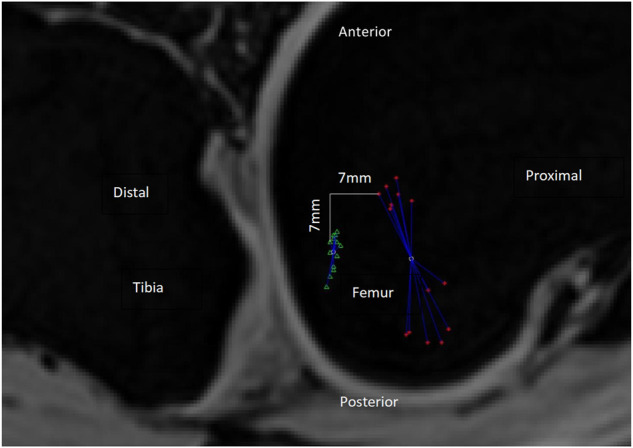
PC value is the standard deviation of the distances between the reference point’s trajectory coordinates and the average coordinate. As shown in the figure, the green triangle’s trajectory is more gathered than that of the red asterisk, so the PC value of the green triangle should be less than that of the red asterisk. The actual calculation result of the PC value is indeed the case [13.7 mm (red asterisk) and 4.5 mm (green triangle), respectively]. The smaller the PC value, the more gathered is the trajectory and the more stable is the reference point during the 0–130° knee flexion. In this Figure 2D distances are used as an illustration, but in the actual experiment, 3D distances are used to calculate the PC value. Red asterisk: the trajectory of the medial condyle of TEA. Green triangle: the trajectory of the reference point 7 mm distal and posterior to the medial condyle of TEA. White circles: there are two white circles, representing the average position of the red asterisk and the green triangle, respectively. Blue line segments: each line segment represents the distance between a reference point coordinate and the average position of all coordinates.

**FIGURE 6:**
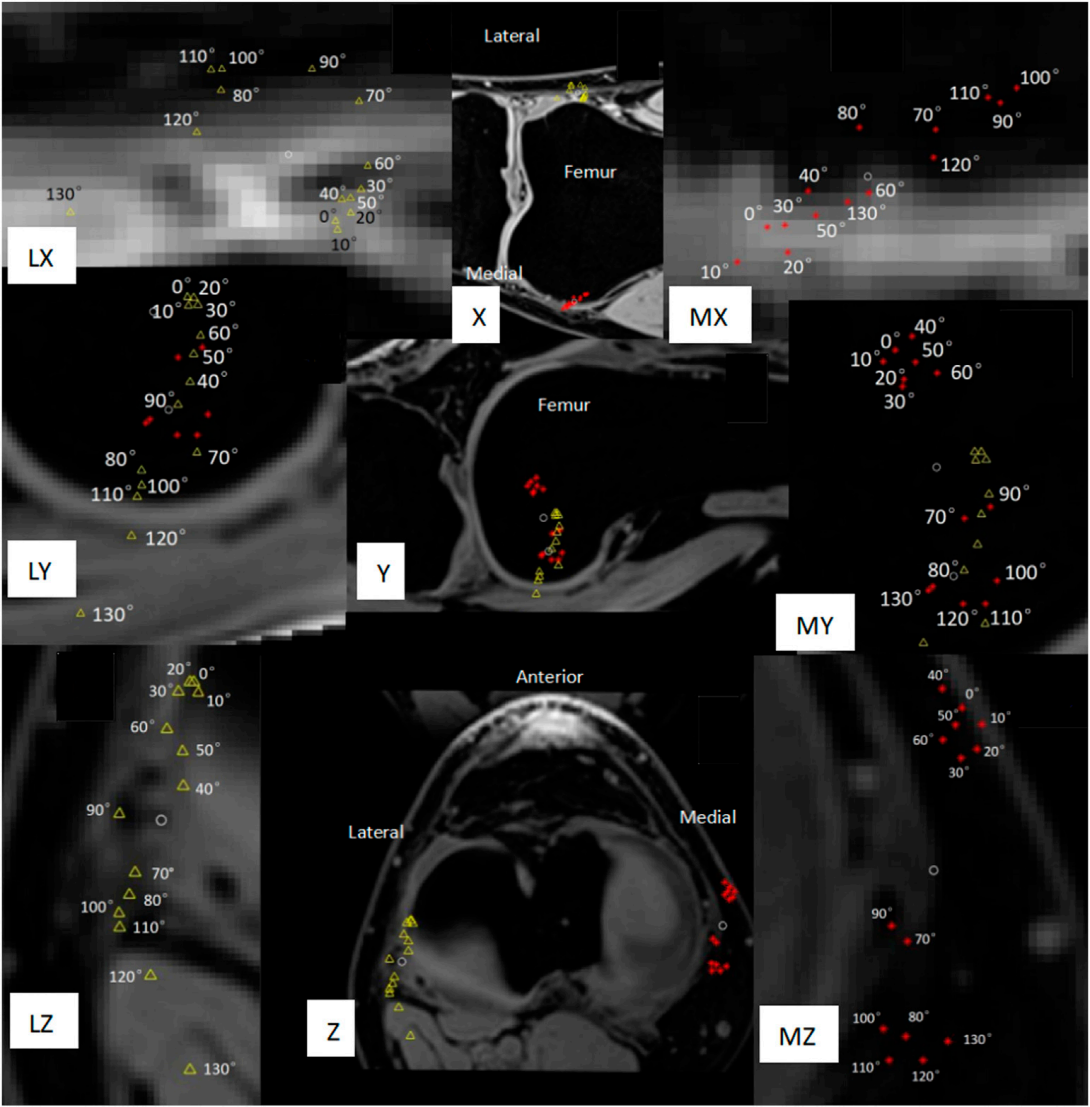
Movement trajectory of the medial/lateral condyle of the femur with the tibia as a fixed rigid body. The red asterisk represents the medial condyle, the yellow triangle represents the lateral condyle, and the two white circles represent the average position of the movement trajectory of the medial condyle and the lateral condyle, respectively. PC is the standard deviation of the distance between the white circle and each red asterisk/yellow triangle around it. (X) Coronal plane. (Y) Sagittal plane. (Z) Horizontal plane. (MX, MY, MZ) Local magnification of the movement trajectory of the femoral medial condyle. (LX, LY, LZ) Local enlargement of the movement trajectory of the femoral lateral condyle. (Z, LZ, MZ) The reverse screw home phenomenon at the flexion of 40°–70° and screw home of flexion over 70° should be noted.

**FIGURE 7:**
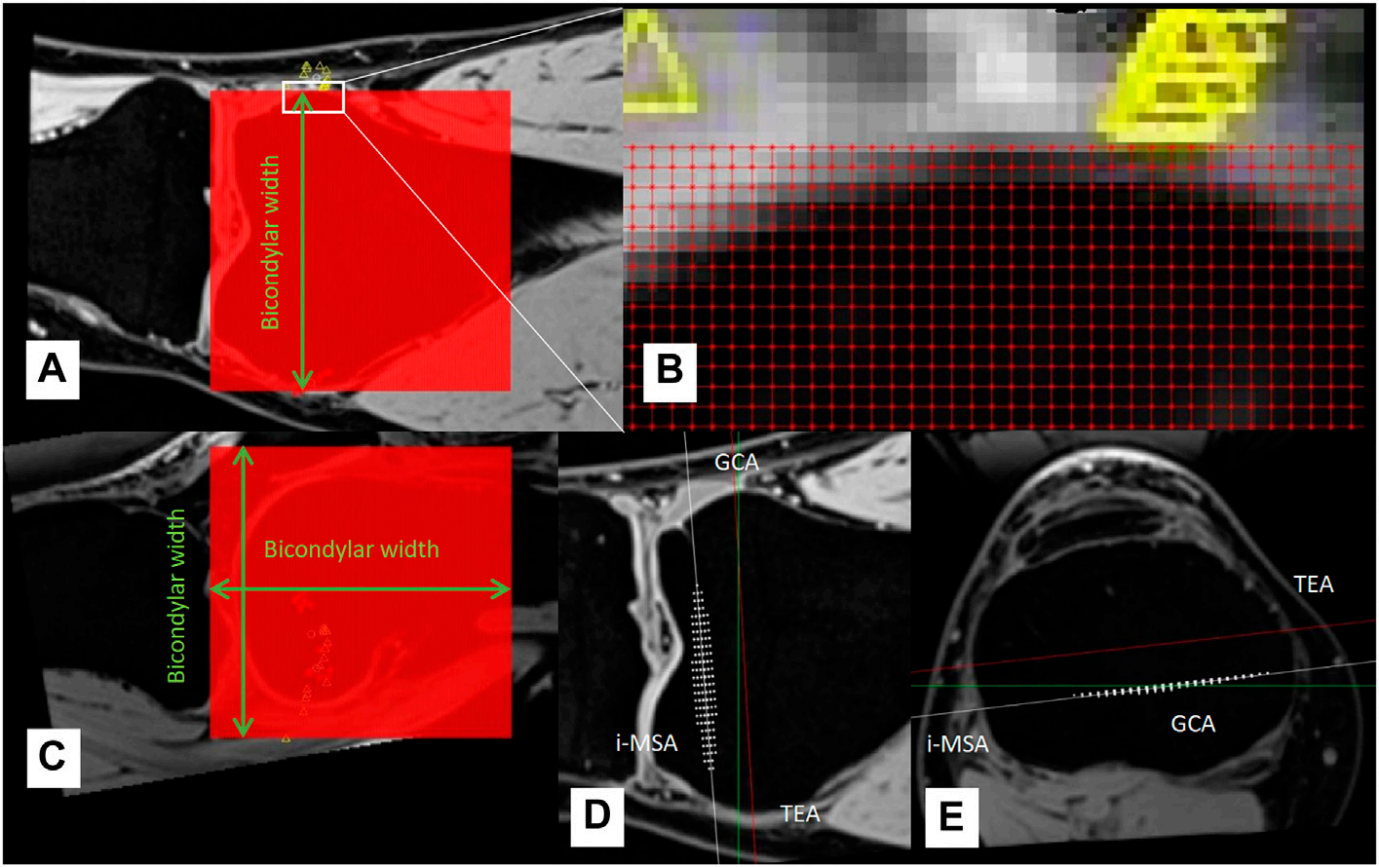
**(A–C)** A cube grid with the side length of bicondylar width was established at the distal end of the femur, the grid spacing was 1 mm, and all grid intersections were candidate reference points. **(D–E)** White asterisks represent the 0.2% reference points with the smallest PC, which was used to determine rotation axis (individual most stable rotation axis, i-MSA).

The tibia was the fixed rigid body and the femur was the moving rigid body. A femur reference point had 14 coordinates during the knee flexion from 0° to 130° (each coordinate was measured/calculated in an MRI image, there are 14 MRI images with knee flexion from 0° to 130° captured at intervals of 10°), and the standard deviation of distance from the average coordinate was used to measure the positional change (PC) of this reference point during the knee flexion from 0° to 130° (Figures 5, 6). Obviously, the smaller the PC of a reference point, the more stable the reference point was during knee movement. We employed the method in Figures 7A–C to determine the range of candidate reference points. Subsequently, we calculated the respective PC of all reference points and selected the 0.2% reference point with the smallest PC value to fit a straight line with the least square method, which was then used as the rotation axis (Figures 7D,E).

### Difference Between/Within the Measurers

In the *in vitro* accuracy test experiment, two musculoskeletal radiologists (SXT and YHL) with over 5 years of experience independently confirmed the CAF of aquarium stones twice, and the interval between the two measurements was more than 1 week. The relative motion between aquarium stones was calculated using MATLAB software. For the accuracy test of θ, V, and V′, an inter-class consistency (ICC) was used to assess the intra-measurer and inter-measurer consistency. The mean ± standard deviation was used to describe their accuracy (Table 1), and their consistency was also evaluated by ICC.

**TABLE 1:** All angles/lengths tested in the experiment have a measurement accuracy of <1°/1 mm; see text for details.

Actual length/angle	Accuracy (95% CI)*^#^	Intra-measurer ICC*	Inter-measurer ICC*
Measurement accuracy of V and θ			
Length (mm)			
1	[0.00,0.00]/[0.00,0.00]/[0.00,0.00]	1.000/1.000/1.0001.000/1.000/1.000	1.000/1.000/1.0001.000/1.000/1.000
2	[0.00,0.00]/[0.00,0.00]/[0.00,0.00]		
5	[0.00,0.00]/[0.00,0.00]/[0.00,0.00]		
10	[0.00,0.00]/[−0.79,0.04]/[−0.68,0.18]		
20	[0.00,0.00]/[0.00,0.00]/[−0.09,0.034]		
50	[−0.61,0.86]/[−0.68,0.18]/[−0.67,042]		
100	[−0.61,0.86]/[−0.68,0.18]/[−0.68,0.18]		
Angle (deg)			
1	[0.00,0.00]/[0.00,0.00]/[0.00,0.00]		
5	[0.00,0.00]/[0.00,0.00]/[0.00,0.00]		
10	[−0.81,0.31]/[0.00,0.00]/[−0.59,−0.16]		
20	[−0.31,0.81]/[0.15,0.85]/[−0.29,0.54]		
50	[−0.09,0.34]/[−1.00,0.00]/[−0.35,0.35]		
80	[−0.18,0.68]/[0.00,1.00]/[−0.18,0.68]		
150	[−0.81,0.68]/[−0.33,0.85]/[−0.67,0.18]		
**Actual length**	Accuracy (95% CI)*∧	Intra-measurer ICC*	Inter-measurer ICC*
Measurement accuracy of V′			
Length (mm)			
1	[−0.02,0.01]/[−0.00,0.02]/[−0.01,0.01]	1.000/1.000/1.000	1.000/1.000/1.000
2	[−0.02,0.01]/[−0.01,0.01]/[−0.02,0.01]		
5	[−0.01,0.02]/[−0.03,0.01]/[−0.02,−0.01]		
10	[−0.01,0.02]/[−0.78,0.04]/[−0.68,0.20]		
20	[−0.00,0.01]/[−0.02,0.01]/[−0.10,0.35]		
50	[−0.63,0.86/[−0.67,0.17]/[−0.69,0.41]		
100	[−0.61,0.87]/[−0.68,0.17]/[−0.69,0.18]		

* Measurement results in different directions are separated by “/,” indicating the results of the coronal plane/sagittal plane/horizontal plane.

^#^Two measurers were measured twice; there are a total of four measurement results used to calculate the mean and standard deviation of the accuracy of V.

^∧^There are a total of 3,374 reference points within the range of 7 cm from V, and there are a total of 4*3,374 = 13,496 measurement results, which are used to calculate the mean and standard deviation of the accuracy V′.

In the *in vivo* experiment, the knee joint CAF was independently confirmed by the same two measurers, and the ICC equaled 1.000. Hence, the results of the measurer SXT were used as the experimental data.

In all experiments, the measurers do not know the exact value of kinematics parameters, they only confirm the CAF. Even if the measurers know the kinematics measurement data, they cannot estimate the position of the reference points P and P′, because it can only be obtained after a lot of calculations. These calculations were performed using MATLAB software, and these calculated results are blind to the measurers.

## Results

### *In Vitro* Measurement Accuracy Test

The accuracy verification results are summarized in Table 1. Overall, all the angles/lengths that were tested in the experiment have a measurement accuracy of <1°/1 mm. It seems that the clinical 3T MRI is sufficient to obtain good accuracy. In addition, the range of angles/lengths validated here is sufficient for knee joint measurements.

### *In Vivo* Experiment

In this study, the knee joints of 36 healthy elderly subjects (14 men and 22 women) were measured. The general information on the subjects is provided in Table 2. Among the 36 measured knee joints, the trajectory of a common medial/lateral femoral condyle is shown in Figure 6. Roll-back motion was observed in all 36 knee joints, and the rollback distance of the medial femoral condyle was 18.1 ± 3.7 mm, and that of the lateral femoral condyle was 31.1 ± 7.3 mm. In addition, the screw home phenomenon was observed in all knee joints, most lateral femoral condyle (21/36, 58%) began to move significantly backward only during knee flexion over 70°.

**TABLE 2:** General information of *in vivo* test subjects.

	Male	Female
Cases	14	22
Age (y)	65.3 ± 9.6 (53–79)	63.5 ± 6.2 (52–77)
Height (cm)	168.6 ± 5.4 (162–179)	160.3 ± 4.1 (152–167)
Weight (kg)	76.1 ± 6.5 (64–87)	63.7 ± 8.2 (52–77)
BMI	26.8 ± 2.8 (23.4–32.4)	24.8 ± 3.2 (20.2–30.0)
Left knee	8 (57%)	10 (45%)

## Discussion

This study proposed a method to describe and measure knee joint kinematics. Our work showed that the rotation angle parameter θ of the rigid body is uniform (Eq. 2 and Figure 2), so the verification of the rotation angle in this study was also uniform. However, the displacement parameter V was not uniform, and the displacement V′ of different reference points P′ was different (Eq. 2 and Figure 2), so the V and V′ needed to be verified separately. Verification experiments showed that the accuracy of the knee joint measurement was <1° and 1 mm with our method. This method can be used to determine the optimal rotation axis of each patient’s knee joint before TKA, prior to the adjustment of prosthesis installation during TKA in a personalized manner, which may improve the surgery effect of TKA and improve the satisfaction rate of patients.

In the “*Materials and Methods*” section we describe a method for determining the optimal axis of rotation for TKA surgery, based on kinematic measurements. Regarding this method, this study focuses on its logic; therefore, the measurement results of the rotation axis are not listed. It is easy to imagine that: 1) if the tibia is used as a fixed rigid body, each reference point on the femur will have its own motion trajectory during knee flexion from 0° to 130°, and the PC of the motion trajectory can be used to measure the stability of this reference point during knee joint movement such that the smaller the PC, the more stable the reference point; 2) strict pivot movement will produce an absolutely stable rotation axis, and the PCs of all reference points on this axis should be 0. In reality, knee joint movement is mainly pivot motion (flexion and extension), accompanied by internal and external rotation, varus-valgus rotation, rollback, screw home, medial pivot, and other unknown movements that may exist. So, the movement of the knee joint is not a strict pivot motion. Although we could not find an absolutely stable rotation axis, in this study, we still observed all the reference points of the distal femur at 1 mm intervals and calculated the PC of each reference point to find which reference points produce a smaller PC. Theoretically, the rotation axis of the femoral prosthesis should be installed in a straight line formed by the reference points with a smaller PC, so that the prosthesis is more stable after TKA and the knee motion is closer to the natural knee.

Our *in vivo* measurements were broadly consistent with those of other researchers, but our results revealed more detail about knee joint movement. Rollback (Figure 6Y, Z) and screw home phenomena (Figure 6Z, LZ, MZ) were observed in our measurements. The measurement results of Tanifuji et al. (2013) showed that the distance of rollback of the medial and lateral ends of transepicondylar axis (TEA) was 18.1 ± 3.7 mm and 31.1 ± 7.3 mm, respectively. Our measurement results were 21.5 ± 4.2 mm and 31.1 ± 7.1 mm, therefore, the data were consistent. In addition, our results showed that the medial end of TEA moved 3.1 ± 1.6 mm to the proximal side and the lateral end moved 5.4 ± 2.9 mm to the distal side during rollback. Kono et al. (2018), Tanifuji et al. (2013) believed that the lateral condyle of TEA moved forward by about 3 mm during knee flexion from 0° to 40°, and moved backward during knee flexion over 40°, which was also similar to our measurement results of the relative movement trajectory of the TEA lateral condyle. However, our results further showed that the most lateral TEA condyle (21/36, 58%) began to move significantly backward only during knee flexion over 70°. These details may help design new prostheses that better conform to the natural knee kinematics.

Although the *in vitro* accuracy verification experiment in this study used aquarium stone as the measurement object, which was not exactly the same as the *in vivo* experiment of knee joint measurement, we believe that this difference does not have a significant impact on the measurement accuracy of angle and length. Because MRI mainly relies on gradient magnetic fields (determine layer) and 2-dimensional Fourier transformation (determine the image for each layer) for localization, they are related to the MRI machine and not related to whether the object being examined is inside or outside the body. The important factor affecting measurement accuracy is the CAF criteria (*In vitro* measurement accuracy test, materials, and methods) preset before the measurement. In this study, the CAF criteria for both *in vivo* and *in vitro* experiments were uniform.

Our method may have some important implications. Currently, there are 3 clinically applied rotational guides for aligning the rotation of the TKA femoral component: TEA, 3° external from the posterior condylar axis (PCA), and Whiteside’s line. These lines, however, are mainly summarized based on the experience of clinicians, which are not necessarily accurate and cannot reflect the individual differences in knee joint rotation axis in each patient. In fact, regarding the rotation alignment of the TKA prosthesis, Poilvache believed that the use of any specific prosthesis alignment technique will result in impaired implant durability or dissatisfaction in some patients, the external rotation angle of the femoral prosthesis appears to be variable and must be adjusted for each knee (Poilvache, 2002). Our method can find the individual most stable rotation axis (i-MSA) (Figure 7), which makes it possible to individually install TKA prostheses in the surgery.

**FIGURE 8:**
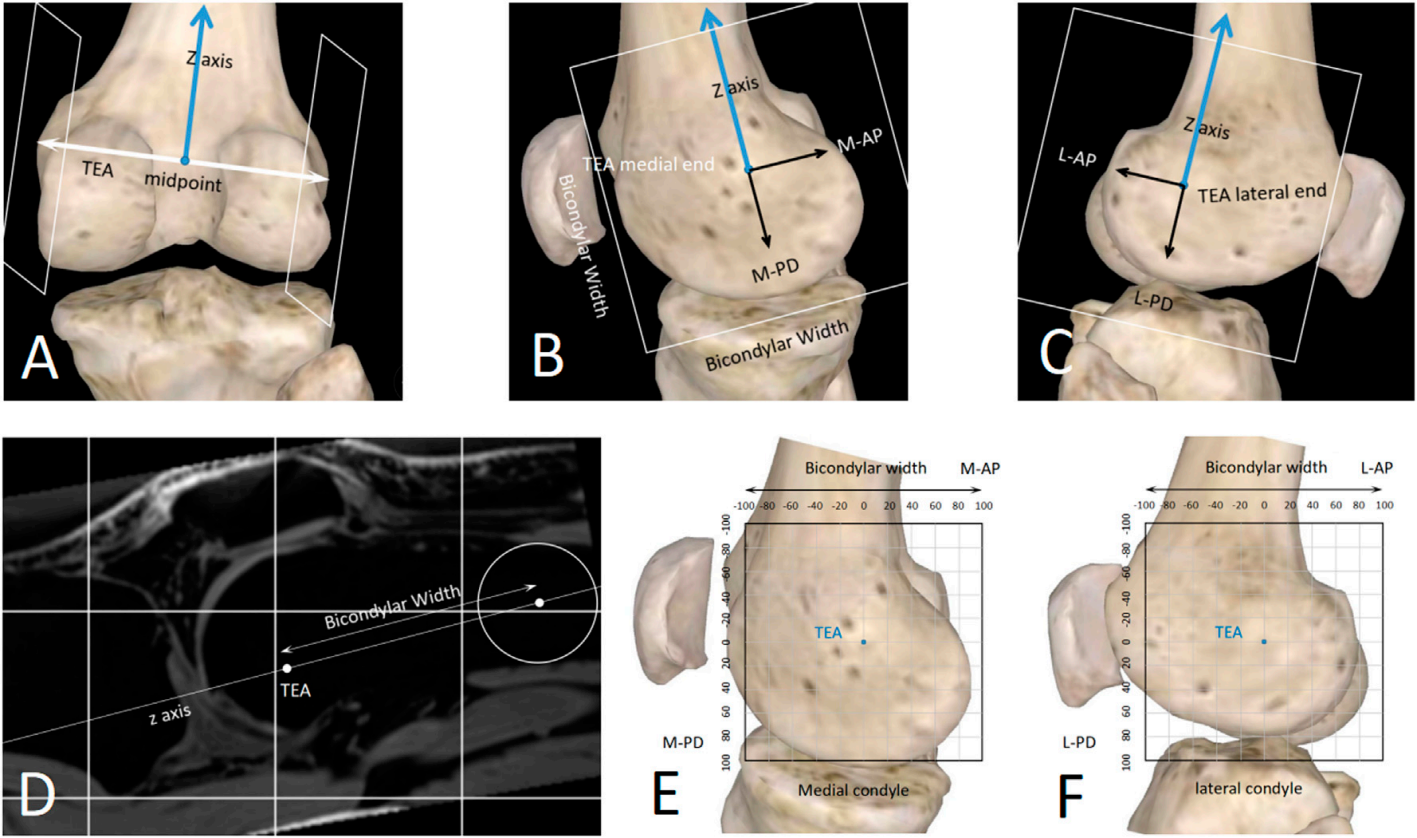
A standardized method to describe spatial position of any straight line close to the knee joint. **(A–D)** The sagittal planes of the TEA medial/lateral ends were determined with the distal femur anatomy. **(E,F)** 2D coordinate systems were established in the sagittal planes. The unit length can be millimeter (sagittal-mm coordinate system) or bicondylar width (sagittal-BCW coordinate system).

Because our method is time-consuming, currently it is difficult to preoperatively measure the i-MSA for every TKA patient. To benefit more TKA patients, we propose the concept of population averaged most stable rotational axis (a-MSA). For TKA patients who have not measured i-MSA, a-MSA is a low-cost alternative that can be considered. We believe that a-MSA should be described in the TEA coordinate system [a similar coordinate system has been proposed by Yu et al. (2021) (Figure 8)] because it is constant in TKA patients (OA does not cause bone defect in TEA). The researchers should have measured the i-MSA of many healthy subjects in advance, then determine the 4 parameters (M-AP, M-PD, L-AP, L-PD) of each subject’s i-MSA in the TEA-coordinate system (Figure 8), and finally, take the average value of each parameter to get the corresponding parameters of a-MSA. For a new patient about to undergo TKA surgery, his or her a-MSA can be determined on preoperative knee MRI according to the 4 parameters of a-MSA. This avoids the time-consuming measurement steps of i-MSA. Further studies will be needed in the future to compare the PC values of a-MSA and conventional rotational axes, and to assess whether a-MSA is more stable than conventional rotational axes. We believe it is less stable than i-MSA but more stable than TEA and GCA.

Interpreting our findings needs caution. Trying to reproduce an individual patient’s kinematics may improve outcomes, but this is not absolute, because clinical outcomes are multifactorial. More research and validation should be performed to continuously refine TKA prosthesis design and their surgical insertion.

This study has some limitations. First, our method currently only measures static knee kinematics. Due to the current level of technological development, MRI examination is time-consuming, so it cannot dynamically record the knee kinematics like X-ray fluoroscopy. But MRI has a unique advantage; it can obtain enough 3-dimensional data to make it possible to find the optimal rotation axis. If the future development of imaging techniques can significantly speed up MRI examinations, our method could be used to measure dynamic knee kinematics. Secondly, our method requires manual confirmation of CAF, so the measurement process is time-consuming for now (It takes 40–50 min to measure a knee joint for a radiologist), which is not conducive to the widespread clinical application of this method. However, a-MSA may be used as a low-cost alternative; it only takes 4–5 min to measure. Thirdly, the study only examined the knee joints of healthy people and did not assess the knee joints of TKA patients with end-stage osteoarthritis. Such knee joints are often combined with bone defects, which may have a significant impact on the knee joint kinematics. However, it is a common defect in current studies, as most studies use healthy knee joints as the measurement object (Asano et al., 2005; Tanifuji et al., 2011; Tanifuji et al., 2013; Kono et al., 2018; Hans et al., 2021). At present, the kinematics of healthy knee joints has not been fully clarified. If the healthy knee joints have been studied thoroughly in the future it may then be necessary to further study the effect of different osteoarthritic grades on knee kinematics.

In conclusion, in the application scenario of knee joint kinematics measurement, our method has an accuracy of <1° of rotation angle and <1 mm of translation for all reference points, and it can be employed to identify the most stable axis of the knee joint.

## Data Availability

The original contributions presented in the study are included in the article/supplementary material; further inquiries can be directed to the corresponding author.

## References

[B1] Asano T. Akagi M. Nakamura T. (2005). The Functional Flexion-Extension Axis of the Knee Corresponds to the Surgical Epicondylar Axis. J. Arthroplasty 20 (8), 1060–1067. 10.1016/j.arth.2004.08.005 16376264

[B2] Baker P. N. Rushton S. Jameson S. S. Reed M. Gregg P. Deehan D. J. (2013). Patient Satisfaction with Total Knee Replacement Cannot Be Predicted from Pre-operative Variables Alone: A Cohort Study from the National Joint Registry for England and Wales. Bone Jt. J. 95-B (95-B), 1359–1365. 10.1302/0301-620X.95B10.32281 24078532

[B3] Beach A. Regazzola G. Neri T. Verheul R. Parker D. (2019). The Effect of Knee Prosthesis Design on Tibiofemoral Biomechanics during Extension Tasks Following Total Knee Arthroplasty. Knee 26 (5), 1010–1019. 10.1016/j.knee.2019.07.008 31402095

[B4] Bruno F. Barile A. Arrigoni F. Laporta A. Russo A. Carotti M. (2018). Weight-bearing MRI of the Knee: a Review of Advantages and Limits. Acta Biomed. 89 (89), 78–88. 10.23750/abm.v89i1-S.7011 PMC6179065 29350638

[B5] Cacciola G. De Martino I. De Meo F. (2020). Does the Medial Pivot Knee Improve the Clinical and Radiographic Outcome of Total Knee Arthroplasty? A Single Centre Study on Two Hundred and Ninety Seven Patients. Int. Orthop. (SICOT) 44 (2), 291–299. 10.1007/s00264-019-04462-3 31865446

[B6] Dennis D. A. Mahfouz M. R. Komistek R. D. Hoff W. (2005). *In Vivo* determination of Normal and Anterior Cruciate Ligament-Deficient Knee Kinematics. J. Biomechanics 38 (2), 241–253. 10.1016/j.jbiomech.2004.02.042 15598450

[B7] Dunbar M. J. Richardson G. Robertsson O. (2013). I Can't Get No Satisfaction after My Total Knee Replacement: Rhymes and Reasons. Bone Jt. J. 95-B (95-B), 148–152. 10.1302/0301-620X.95B11.32767 24187375

[B8] Dzialo C. M. Pedersen P. H. Simonsen C. W. Jensen K. K. de Zee M. Andersen M. S. (2018). Development and Validation of a Subject-specific Moving-axis Tibiofemoral Joint Model Using MRI and EOS Imaging during a Quasi-Static Lunge. J. Biomechanics 72 (27), 71–80. 10.1016/j.jbiomech.2018.02.032 29567307

[B9] Eckhoff D. Hogan C. DiMatteo L. Robinson M. Bach J. (2007). An Abjs Best Paper. Clin. Orthop. Relat. Res. 461 (461), 238–244. 10.1097/blo.0b013e318112416b 17549027

[B10] Fitzpatrick C. K. Maag C. Clary C. W. Metcalfe A. Langhorn J. Rullkoetter P. J. (2016). Validation of a New Computational 6-DOF Knee Simulator during Dynamic Activities. J. Biomech. 49 (49), 3177–3184. 10.1016/j.jbiomech.2016.07.040 27545078

[B11] Freeman M. A. R. Pinskerova V. (2005). The Movement of the Normal Tibio-Femoral Joint. J. Biomechanics 38 (2), 197–208. 10.1016/j.jbiomech.2004.02.006 15598446

[B12] Giphart J. E. Zirker C. A. Myers C. A. Pennington W. W. LaPrade R. F. (2012). Accuracy of a Contour-Based Biplane Fluoroscopy Technique for Tracking Knee Joint Kinematics of Different Speeds. J. Biomechanics 45 (16), 2935–2938. 10.1016/j.jbiomech.2012.08.045 23021610

[B13] Gogia P. P. Braatz J. H. Rose S. J. Norton B. J. (1987). Reliability and Validity of Goniometric Measurements at the Knee. Phys. Ther. 67 (2), 192–195. 10.1093/ptj/67.2.192 3809242

[B14] Guan S. Gray H. A. Schache A. G. Feller J. de Steiger R. Pandy M. G. (2017). *In Vivo* six-degree-of-freedom Knee-Joint Kinematics in Overground and Treadmill Walking Following Total Knee Arthroplasty. J. Orthop. Res. 35 (35), 1634–1643. 10.1002/jor.23466 27770609

[B15] Hans A. G. Guan S. Thomeer T. L. Pandy M. G. (2021). Moment Arm of the Knee-Extensor Mechanism Measured *In Vivo* across a Range of Daily Activities. J. Biomechanics 123, 110484. 10.1016/j.jbiomech.2021.110484 34062347

[B16] Hollister A. M. Jatana S. Singh A. K. Sullivan W. W. Lupichuk A. G. (1993). The Axes of Rotation of the Knee. Clin. Orthop. Relat. Res. 290, 2259–2268. 10.1097/00003086-199305000-00033 8472457

[B17] Iranpour F. Merican A. M. Baena F. R. Y. Cobb J. P. Amis A. A. (2010). Patellofemoral Joint Kinematics: The Circular Path of the Patella Around the Trochlear axis. J. Orthop. Res. 28 (5), 589–594. 10.1002/jor.21051 19950364

[B18] Justin D. B. Petruccelli D. Adili A. Piccirillo L. Wismer D. Winemaker M. (2012). Patient Perspective Survey of Total Hip vs Total Knee Arthroplasty Surgery. J. Arthroplasty 27 (6), 865–869. e5. 10.1016/j.arth.2011.12.031 22333864

[B19] Kono K. Tomita T. Futai K. Yamazaki T. Tanaka S. Yoshikawa H. (2018). *In Vivo* three-dimensional Kinematics of Normal Knees during Different High-Flexion Activities. Bone & Jt. J. 100-B (1100), 50–55. 10.1302/0301-620x.100b1.bjj-2017-0553.r2 PMC5842511 29305450

[B20] Kurtz S. Ong K. Lau E. Mowat F. Halpern M. (2007). Projections of Primary and Revision Hip and Knee Arthroplasty in the United States from 2005 to 2030. J. Bone & Jt. Surg. 89 (4), 780–785. 10.2106/jbjs.f.00222 17403800

[B21] Li G. Kozanek M. Hosseini A. Liu F. Van de Velde S. K. Rubash H. E. (2009). New Fluoroscopic Imaging Technique for Investigation of 6DOF Knee Kinematics during Treadmill Gait. J. Orthop. Surg. Res. 4 (13), 6. 10.1186/1749-799X-4-6 19284658 PMC2669052

[B22] Meneghini R. M. Deckard E. R. Ishmael M. K. Ziemba-Davis M. (2017). A Dual-Pivot Pattern Simulating Native Knee Kinematics Optimizes Functional Outcomes after Total Knee Arthroplasty. J. Arthroplasty 32 (32), 3009–3015. 10.1016/j.arth.2017.04.050 28648702

[B23] Mohsen A. S. Boulos A. Saevarsson S. K. Yoo S. Miller S. Anglin c. (2016). Changes in Knee Kinematics Following Total Knee Arthroplasty. Proc. Inst. Mech. Eng. H. 4 (230), 265–278. 10.1177/0954411916632491 26936959

[B24] Most E. Axe J. Rubash H. Li G. (2004). Sensitivity of the Knee Joint Kinematics Calculation to Selection of Flexion Axes. J. Biomechanics 37 (11), 1743–1748. 10.1016/j.jbiomech.2004.01.025 15388317

[B25] Park A. Duncan S. T. Nunley R. M. Keeney J. A. Barrack R. L. Nam D. (2014). Relationship of the Posterior Femoral axis of the "kinematically Aligned" Total Knee Arthroplasty to the Posterior Condylar, Transepicondylar, and Anteroposterior Femoral Axes. Knee 21 (21), 1120–1123. 10.1016/j.knee.2014.07.025 25112211 PMC4267996

[B26] Poilvache P. (2002). The Epicondylar Axis for Femoral Component Rotation. Surgical Techniques in Total Knee Arthroplasty. New York: Springer.

[B27] Ramsey D. K. Wretenberg P. F. (1999). Biomechanics of the Knee: Methodological Considerations in the *In Vivo* Kinematic Analysis of the Tibiofemoral and Patellofemoral Joint. Clin. Biomech. 14 (9), 595–611. 10.1016/s0268-0033(99)00015-7 10521643

[B28] Reddy N. P. Askew M. J. Baniewicz F. M. Melby A. Fuller K. A. Steurer P. A. (1989). A Technique for Quantitative Assessment of Three-Dimensional Motion with Applications to Human Joints. Proc. Inst. Mech. Eng. H. 203 (4), 207–213. 10.1243/pime_proc_1989_203_041_01 2701958

[B29] Şahin N. Atıcı T. Kurtoğlu U. Turgut A. Ozkaya G. Ozkan Y. (2013). Centre of the Posterior Cruciate Ligament and the Sulcus between Tubercle Spines Are Reliable Landmarks for Tibial Component Placement. Knee Surg. Sports Traumatol. Arthrosc. 21 (10), 2384–2391. 10.1007/s00167-012-2120-5 22751944

[B30] Scarvell J. M. Smith P. N. Refshauge K. M. Galloway H. R. Woods K. R. (2004). Comparison of Kinematic Analysis by Mapping Tibiofemoral Contact with Movement of the Femoral Condylar Centres in Healthy and Anterior Cruciate Ligament Injured Knees. J. Orthop. Res. 22 (22), 955–962. 10.1016/j.orthres.2003.12.016 15304265

[B31] Schache A. G. Baker R. Lamoreux L. W. (2006). Defining the Knee Joint Flexion-Extension axis for Purposes of Quantitative Gait Analysis: An Evaluation of Methods. Gait Posture 24 (1), 100–109. 10.1016/j.gaitpost.2005.08.002 16191481

[B32] Schulze A. Scharf H. P. (2013). Zufriedenheit nach Knietotalendoprothesenimplantation. Orthopäde 42 (10), 858–865. 10.1007/s00132-013-2117-x 23695195

[B33] Shih K.-S. Lin C.-C. Lu H.-L. Fu Y.-C. Lin C.-K. Li S.-Y. (2020). Patient-specific Instrumentation Improves Functional Kinematics of Minimally-Invasive Total Knee Replacements as Revealed by Computerized 3D Fluoroscopy. Comput. Methods Programs Biomed. 188 (188), 105250. 10.1016/j.cmpb.2019.105250 31838341

[B34] Sta S. Ogor J. Letissier H. Stindel E. Hamitouche C. Dardenne G. (2021). Towards Markerless Computer Assisted Surgery: Application to Total Knee Arthroplasty. Int. J. Med. Robot. 17 (5), e2296. 10.1002/rcs.2296 34085387

[B35] Takashi S. Mochizuki T. (2021). Three-dimensional Morphology of the Distal Femur Based on Surgical Epicondylar axis in the Normal Elderly Population. Knee 30, 125–133. 10.1016/j.knee.2021.03.022 33895611

[B36] Tanifuji O. Sato T. Kobayashi K. Mochizuki T. Koga Y. Yamagiwa H. (2011). Three-dimensional *In Vivo* Motion Analysis of Normal Knees Using Single-Plane Fluoroscopy. J. Orthop. Sci. 16 (16), 710–718. 10.1007/s00776-011-0149-9 21892788

[B37] Tanifuji O. Sato T. Kobayashi K. Mochizuki T. Koga Y. Yamagiwa H. (2013). Three-dimensional *In Vivo* Motion Analysis of Normal Knees Employing Transepicondylar axis as an Evaluation Parameter. Knee Surg. Sports Traumatol. Arthrosc. 21 (10), 2301–2308. 10.1007/s00167-012-2010-x 22543470

[B38] Tupling S. J. Pierrynowski M. R. (1987). Use of Cardan Angles to Locate Rigid Bodies in Three-Dimensional Space. Med. Biol. Eng. Comput. 25 (25), 527–532. 10.1007/BF02441745 3446974

[B39] Van Campen A. De Groote F. Bosmans L. Scheys L. Jonkers I. De Schutter J. (2011). Functional Knee axis Based on Isokinetic Dynamometry Data: Comparison of Two Methods, MRI Validation, and Effect on Knee Joint Kinematics. J. Biomechanics 44 (15), 2595–2600. 10.1016/j.jbiomech.2011.08.022 21924426

[B40] Yamazaki T. Futai K. Tomita T. Sato Y. Yoshikawa H. Tamura S. (2015). 3D Kinematics of Mobile-Bearing Total Knee Arthroplasty Using X-Ray Fluoroscopy. Int. J. Comput. Assist. Radiol. Surg. 10 (10), 487–495. 10.1007/s11548-014-1093-x 24965187

[B41] Yu Z. Cai H. Yang B. Yao J. Zhang K. Tian H. (2021). Relationship between Patellofemoral Finite Helical axis and Femoral Trans-epicondylar axis Using a Static Magnetic Resonance-Based Methodology. J. Orthop. Surg. Res. 16 (16), 212. 10.1186/s13018-021-02328-2 33761974 PMC7988974

